# Supplementary material for: Sustainable-use marine protected areas to improve human nutrition
Source: Nat Commun. 2024 Sep 17;15:7716. doi: 10.1038/s41467-024-49830-9 (PMC11408491; doi:10.1038/s41467-024-49830-9)
Supplement: Supplementary file 1 — Supplementary Information [file 41467_2024_49830_MOESM1_ESM.pdf]

Supplementary text

Social, physical, and ecological drivers

Social drivers

Human population (Yeager et al 2017): Human population size surrounding coral reefs based on the Gridded Population of the World, Population Count Grid v3 (GPWv3; CISEN 2005) and Gridded Population of the World v4 (GPWv4) Population Count Adjusted to Match 2015 Revision of UN WPP Country Totals (CISEN 2016) datasets produced by the Socioeconomic Data and Applications Center (SEDAC). To estimate the human population count within 20 km and 50 km radii of each grid cell, we created buffers of the corresponding radius from the mid-point of each grid cell from our base raster layer projected in an azimuthal equal-area projection. We then extracted all grid cells within the corresponding SEDAC population layer that fell within the buffer and summed the population counts within the extracted cells. We report the data as total human population count within a given radius, but data could be converted to average population density by dividing by 1,256 km^2 or 7,850 km^2 for the 20 km and 50 km buffers, respectively.

Market distance (Yeager et al 2017): Distance to provincial capital, a proxy for distance to market and thus fishing intensity, was calculated as the shortest geodesic distance of each survey site to the nearest provincial or national capital. We calculated it using the Near tool in ArcGIS 10.2.2. These data were sourced from the World Cities base map layer provided by ESRI38 (Version 10.1), which also includes major population centers and landmark cities.

Human Development Index: World Development Indicators (WDI) is the primary World Bank collection of development indicators, compiled from officially recognized international sources. It presents the most current and accurate global development data available, and includes national, regional and global estimates. The HDI measures the average achievements in a country in three basic dimensions of human development: a long and healthy life, access to knowledge and a decent standard of living (World Bank).

Fishing pressure: Total number of coral reef fishers divided by coral reef area within each country. Total number of fishers are estimated based on coral reef area, rural coastal population, and fishing pressure and the proportion of reef-related to total marine fish landed values to the total number of marine fishers in a country (Teh et al 2013). Coral reef area within each country was calculated by overlaying reef polygons with each countries' EEZs.

Government effectiveness: Aggregate and individual governance indicators for six dimensions of governance: Voice and Accountability; Political Stability and Absence of Violence/Terrorism; Government Effectiveness; Regulatory Quality; Rule of Law; Control of Corruption (World Bank).

Fisheries management effectiveness: Effectiveness with which fisheries are being managed. Based on averaged scores on the scales of scientific robustness, policymaking transparency, implementation capability, fishing capacity, subsidies, and access to foreign fishing. This study used a survey with over 13,000 fisheries experts to assess the effectiveness of fisheries management regimes worldwide in 2008 (Mora et al 2009).

<u>Physical drivers</u>

Reef area (Yeager et al 2017): For global layers, we calculated coral reef connectivity as the total amount of coral reef area within the surrounding landscape at two buffer distances: 15 km and 200 km. The 15 km buffer distance represents the upper range of adult home range movement distances for most reef fishes, which are largely constrained to 5-15 km (Green et al., 2015). The larger buffer distance of 200 km corresponds to the upper end of larval dispersal distances and home range size for large-bodied species (although longer dispersal distances are possible, but rare, Green et al. 2015). Reef area was estimated from the 500 m resolution coral reef map from the Reefs at Risk Revisited data set created by the World Resources Institute (Burke et al. 2011). For our global grid, area estimates were made by creating a buffer of the corresponding size (15 km or 200 km) around the midpoint of each grid cell using the 'gBuffer' function from the rgeos package (Bivand and Rundel, 2016) projected to the Cylindrical Equal-Area (Lambert) projection (Central Meridian: -160. Datum: WGS 1984; same projection as reef layer) in R (R Core Team 2016). The reef layer was then clipped to the buffer using the 'extract' function in the raster package (Hijmans, 2016a) and the area in km2 estimated as the number of reef cells falling within the buffer multiplied by the area of a cell (0.25 km2). For the MSEC online platform, buffers will be drawn from the user-input geographic coordinates and user can specify their own buffer distance at 1 km intervals ranging from 1 to 200 km.

Land cover (Yeager et al 2017): Terrestrial nutrient and energy flows into marine ecosystems may impact local productivity and food web structure. Previous studies have found that nutrient inputs from land-derived sources are commonly detectable within primary producers with up to 15 km from shore (Lapointe and Clark 1992). However, riverine plumes may bring terrestrial-derived dissolved organic nutrients 50 km or more from the coast (Delvin and Brodie 2005). In most cases, effects of terrestrial sediment run-off are limited to within ~10 km of shore (Fabricius 2005, Devlin and Brodie 2005). Therefore we calculated the amount of land area within two buffer distances (15 km and 50 km) as one metric of the magnitude of terrestrial subsidies into marine ecosystems. Similar to reef area estimates, a buffer around the midpoint of each raster cell was drawn in azimuthal equidistant projection centered on the point (to give true distances) and then projected to the WGS84 geographic coordinates system (coordinate system of the land area raster). We then extracted and summed the area of all land-based grid cells (from the 0.25 arcmin, GSHHS-derived land raster) within the buffer with the 'area' and 'extract' functions of the raster package. As with reef area, user may specify their own buffer distance for land area at 1 km intervals ranging from 1 to 200 km in the MSEC online platform.

Shore distance: Distance to shore represents the shortest geodesic distance to land, using the full resolution shoreline layer of the Global Self-consistent, Hierarchical, High-resolution Geography (GSHHG) v2.3.3 global shoreline dataset.

Wave exposure (Yeager et al 2017): The WAVEWATCH III hindcast dataset (http://polar.ncep.noaa.gov/waves/CFSR_hindcast.shtml) is the product of the namesake wave forecasting model based on wind input from the National Centers for Environmental Prediction's Climate Forecast System Reanalysis (CFSR) (Chawla et al., 2013). The significant height and peak period and direction of waves, as well as the speed and direction of wind, is available at a 3 hour temporal resolution for a span of 31 years (1979-2009). It is composed of 14 nested grids including a 30 arcmin global grid, along with 10 arcmin and 4 arcmin grids for specific coastal areas.

Ecoregions: Marine Ecoregions of the World (MEOW) classification system that defines 232 marine ecoregions. The MEOW system is a biogeographic classification of the world's coasts and shelves (Spalding et al 2007).

<u>Environmental drivers</u>
Chlorophyll: mean levels of chlorophyll in mg/m3 for the surface water layer. The data are available for global-scale applications at a spatial resolution of 5 arcmin (BioOracle).

Photosynthetic active radiation: mean levels of photosynthetically active radiation (PAR) in E/m2/year for the surface water layer. The data are available for global-scale applications at a spatial resolution of 5 arcmin (BioOracle).

Nitrate: mean levels of nitrate in μmol/m3 for the surface water layer. The data are available for global-scale applications at a spatial resolution of 5 arcmin (BIoOracle).

Sea Surface Temperature mean: mean levels of temperature in degrees Celsius (°C) for the surface water layer. The data are available for global-scale applications at a spatial resolution of 5 arcmin (BioOracle).

Sea Surface Temperature minimum: minimum levels of temperature in degrees Celsius (°C) for the surface water layer. The data are available for global-scale applications at a spatial resolution of 5 arcmin (BioOracle).

Sea Surface Temperature range: temperature range in degrees Celsius (°C) for the surface water layer. The data are available for global-scale applications at a spatial resolution of 5 arcmin.

Surplus production fisheries model

We used a surplus production model to estimate catch from biomass predictions in each coral reef polygon. Surplus production models can be used to describe stock status and exploitation in data-limited fisheries (Schaefer, 1954; Costello et al 2016; Punt, 2003; Beverton

and Holt, 1957). They assume that sustainable catch is a simple function of population biomass, regardless of the size and age composition of that biomass (Holt, 2014). In addition, they assume that the population is at equilibrium and that productivity is constrained by a constant carrying capacity of the environment. The most widely used surplus production model is the one developed by Schaefer (1954):

$$B_{t+1} = B_t + B_t * r * \left(1 - \frac{Bt}{K}\right) - Yt$$

Where *B* is the biomass of the species or population at time *t* and *t+1*, *r* is the intrinsic rate of population growth, *K* is the carrying capacity of the environment, and *Y* is the catch at time *t*. Under the Schaefer model, B_{MMSY} = K/2, F_{MMSY} = r/2, and MMSY=rK/4.

Assuming B_{MMSY} as a reference point, we can estimate K as K=2*B_{MMSY}. Therefore, for any given biomass level, the sustainable harvest rate will be equal to the growth of the population represented by $h_i = r * \left(1 - \frac{B_i}{K}\right)$

Therefore, predicted catch for reef *i* is:

$$Y_i = h_i * B_i$$

Catch (Y) per species (j) is based on the catch proportion (α) of coral reef fisheries from the Sea Around Us project (SAU):

$$Y_{i,j} = \alpha_j * Y_i$$

Nutrient supply

The supply of nutrient (k) in reef *i* is:

$$S_{k,i} = \sum_{j=1}^{j} \left(Y_{i,j} * \delta_{j,k}\right)$$

Where $S_{k,i}$is the total supply of nutrient *k* from reef *i* and $\delta_{k,j}$is the nutrient composition of species *j*.

Per capita nutrient supply P_i is then:

$$P_i = \sum_{i=1}^{i} \left(S_{k,i}\right) / \sum_{i=1}^{i} (N_i)$$

Where N is the total number of people around reef i.

Propagating uncertainty

We used Monte Carlo simulation to propagate uncertainty across all steps of the analysis. We ran 10,000 iterations of the model and for each iteration we generated a random value for each parameter in the model based on a known parameter distribution. For population growth rate,

we used a normal distribution with a mean of 0.23 and a standard deviation of 0.06. For Bmsy, we generated random numbers for the biomass quantile (see methods for details) between 0.7 and 0.98. To account for uncertainty on the nutritional content of each species we generated a random nutritional value for each species using the mean and standard deviation generated in the species matching process. For example, when a particular species is matched at the family level, the value is drawn from several observations within that family allowing calculation of the mean and standard deviation of each particular match. We then used these calculated values to generate random numbers based on a normal distribution. For population sizes, we randomly selected a buffer the reefs from 5km, 10km, 15km, 20km, 25km or 30km. Figure S11 shows the distribution of all parameter values used in the Monte Carlo simulation, Figure S9 shows the range of results given parameter uncertainty for all nutrients combined and Figure S10 shows the range of results for each nutrient assessed.

Supplementary Tables and Figures

Supplementary Tab. 1 - Social-ecological drivers of reef fish density (kg/ha) considered and retained in the predictive model (SST=sea surface temperature).

#	Covariate	Included?	Source
Environmental			
1	Chlorophyll concentration (mg/m^3)	Included	https://www.bio-oracle.org/
2	Nitrate concentration (μmol/m^3)	Included	https://www.bio-oracle.org/
3	Photosynthetic active radiation (E/m^2/yr)	Included	https://www.bio-oracle.org/
4	SST mean (°C)	Included	https://www.bio-oracle.org/
5	SST range (°C)	Included	https://www.bio-oracle.org/
	SST min (°C)	Excluded	https://www.bio-oracle.org/
	Primary productivity (g/m^3/day)	Excluded	https://www.bio-oracle.org/
Physical			
6	Reef area (km^2)	Included	Yeager et al. 2017
7	Shore distance (km)	Included	Yeager et al. 2017
8	Wave exposure (kW/m)	Included	Yeager et al. 2017
Social			
9	Human population size	Included	Yeager et al. 2017
10	Market distance (km)	Included	Yeager et al. 2017
11	Human development index	Included	http://hdr.undp.org
12	Fisheries management effectiveness	Included	Mora et al. 2009
13	Sustainable-use MPA (MPA)	Included	https://reeflifesurvey.com/
14	Government effectiveness	Included	http://hdr.undp.org
	Fishing pressure (fishers/km^2)	Excluded	Teh et al. 2013
	Land cover (km^2)	Excluded	Yeager et al. 2017
Interaction term			
15	MPA:Fisheries Management Effectiveness	Included	
Random effect (intercept)			
16	Ecoregion	Included	Spalding et al. 2007

Supplementary Tab. 2 - Regions for the countries represented in the study.

Southeast Asia & Pacific	Latin America & Caribbean	Middle East & North Africa	South Asia	Sub-Saharan Africa
Australia	Antigua & Barbuda	Djibouti	Bangladesh	Kenya
Cambodia	Bahamas	Egypt	India	Madagascar
China	Barbados	Iran	Maldives	Mauritius
Fiji	Belize	Israel	Sri Lanka	Mozambique
Indonesia	Brazil	Jordan		South Africa
Japan	Colombia	Kuwait		Tanzania
Kiribati	Costa Rica	Oman		
Malaysia	Cuba	Saudi Arabia		
Myanmar	Dominica	United Arab Emirates		
Philippines	Dominican Republic	Yemen		
Samoa	Ecuador			
Solomon Islands	Grenada			
Taiwan	Haiti			
Thailand	Honduras			
Timor-Leste	Jamaica			
Vanuatu	Mexico			
Vietnam	Nicaragua			
	Panama			

St. Kitts & Nevis

St. Lucia

St. Vincent & Grenadines

Trinidad & Tobago

Venezuela

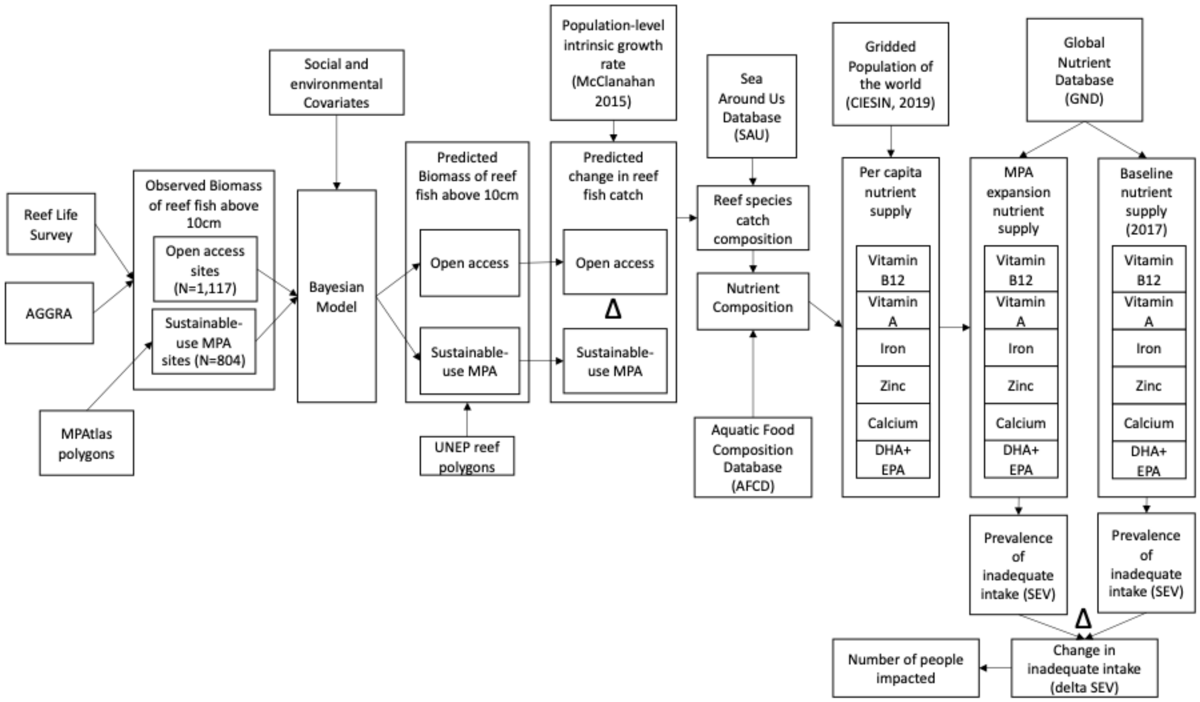

Fig. M1 - Illustration of analysis workflow.

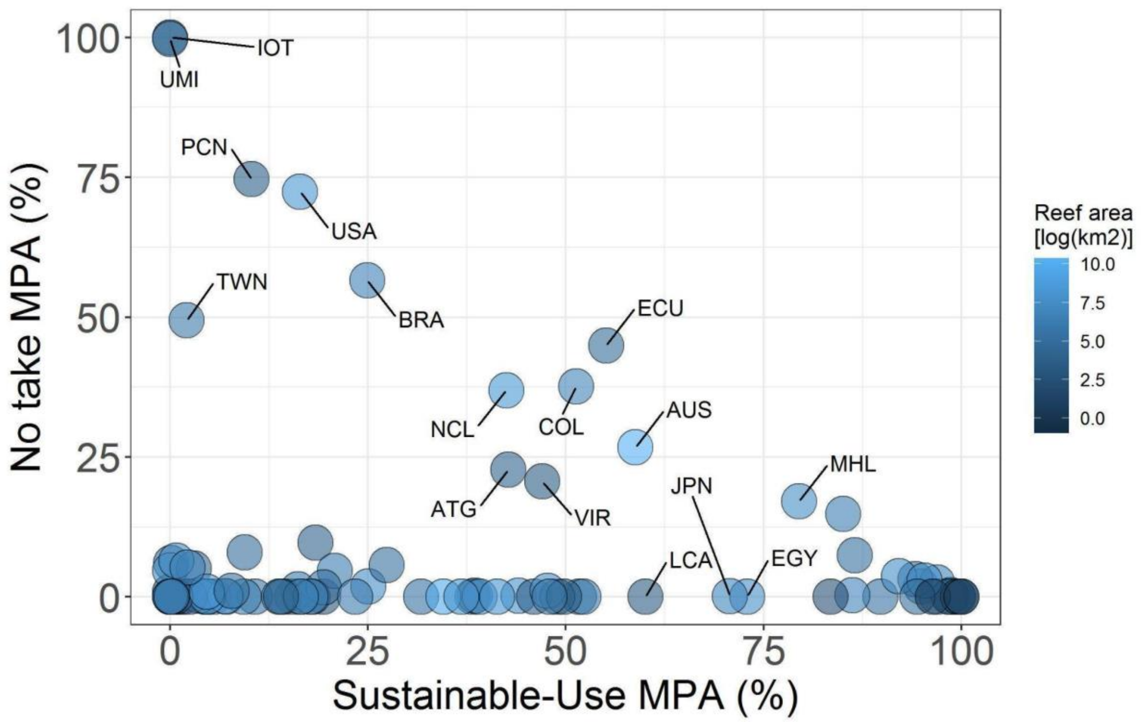

Fig. S1 - Percent of coral reefs within reported sustainable-use and no-take MPAs. Each point represents a country. Three-letter abbreviations represent selected alpha-3 ISO country codes.

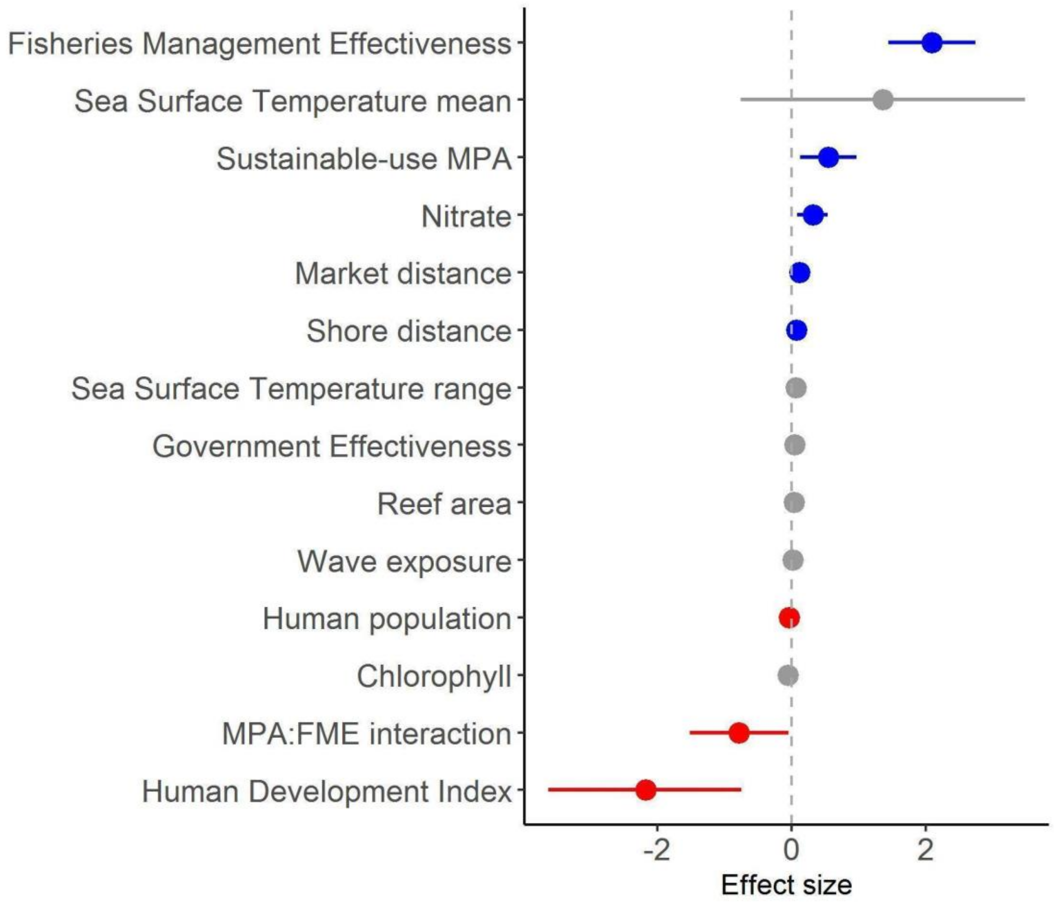

Fig. S2 - Model effect size of social, environmental, and physical covariates. Blue points represent variables with significant positive effects, red points represent variables with significant negative effects, and gray points represent variables that are not significant. “MPA” refers to “Sustainable-use MPAs” and “FME” refers to “Fisheries Management Effectiveness”.

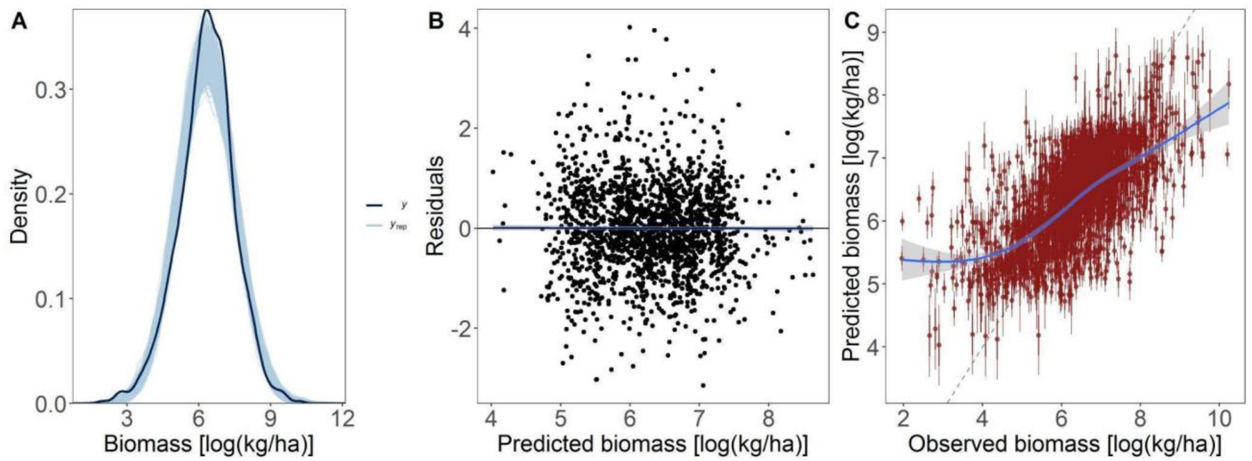

Fig. S3 - Model performance indicators, where (A) are the posterior distributions, (B) are the residuals versus predicted log biomass, and (C) are fitted versus observed values.

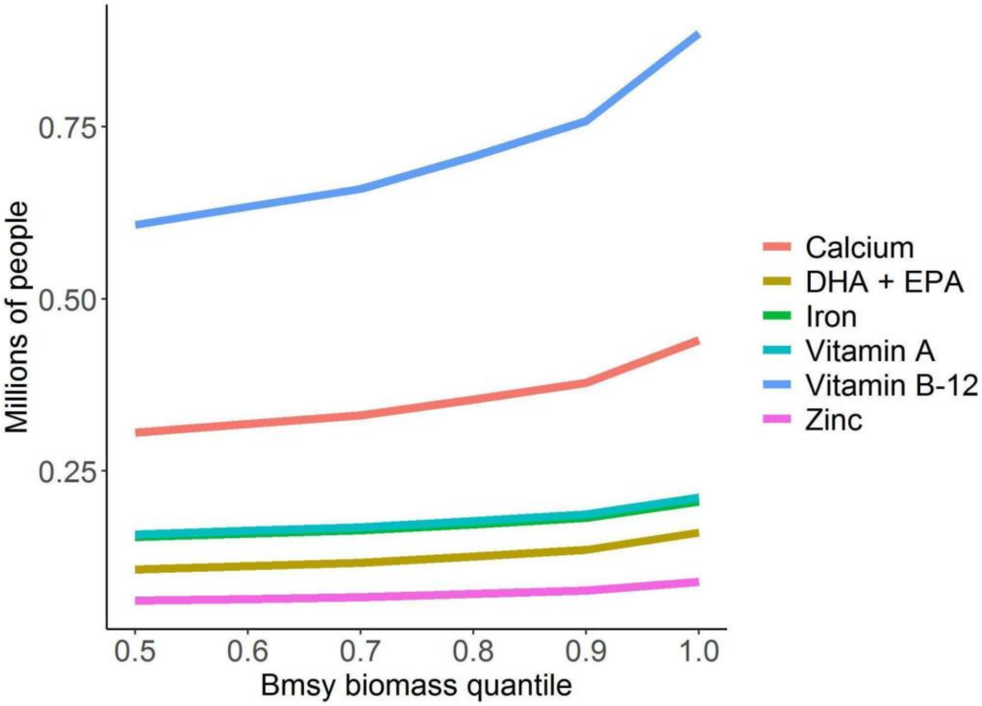

Fig. S4 - Sensitivity of main results to the assumed B_{msy}, which is predicted based on the 90th biomass quantile of highly effective sustainable-use MPA sites. The Y axis represents the total number of people transitioning from inadequate to adequate nutrient intake.

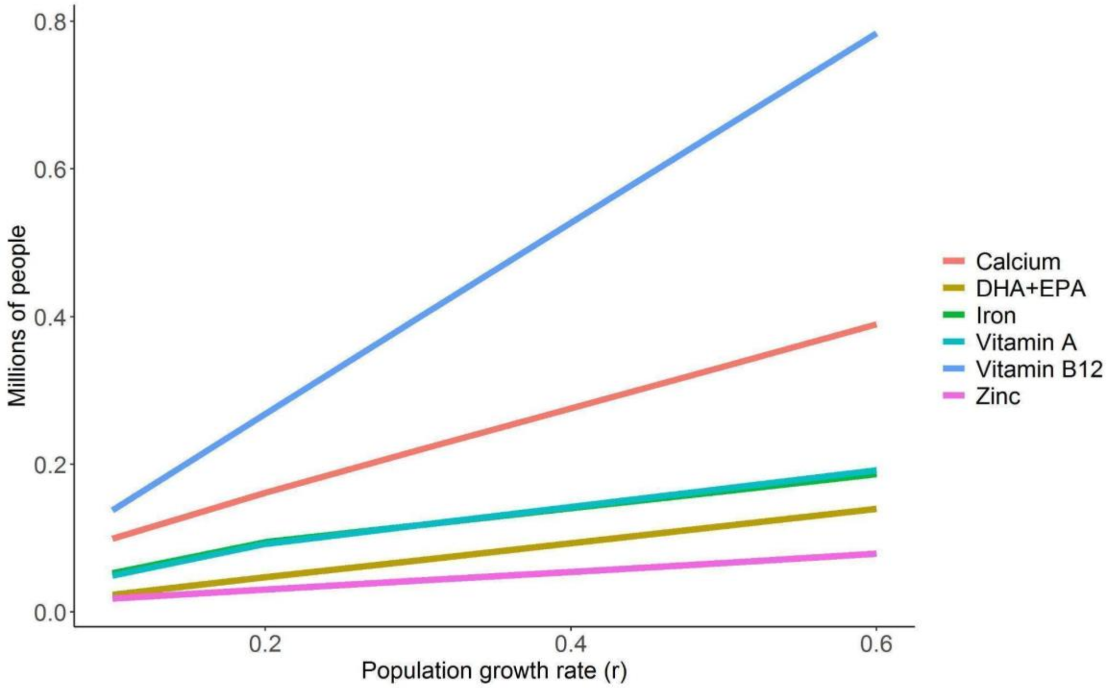

Fig. S5 - Sensitivity of main results to the assumed intrinsic population growth rate (r). The Y axis represent the total number of people transitioning from inadequate to adequate nutrient intake.

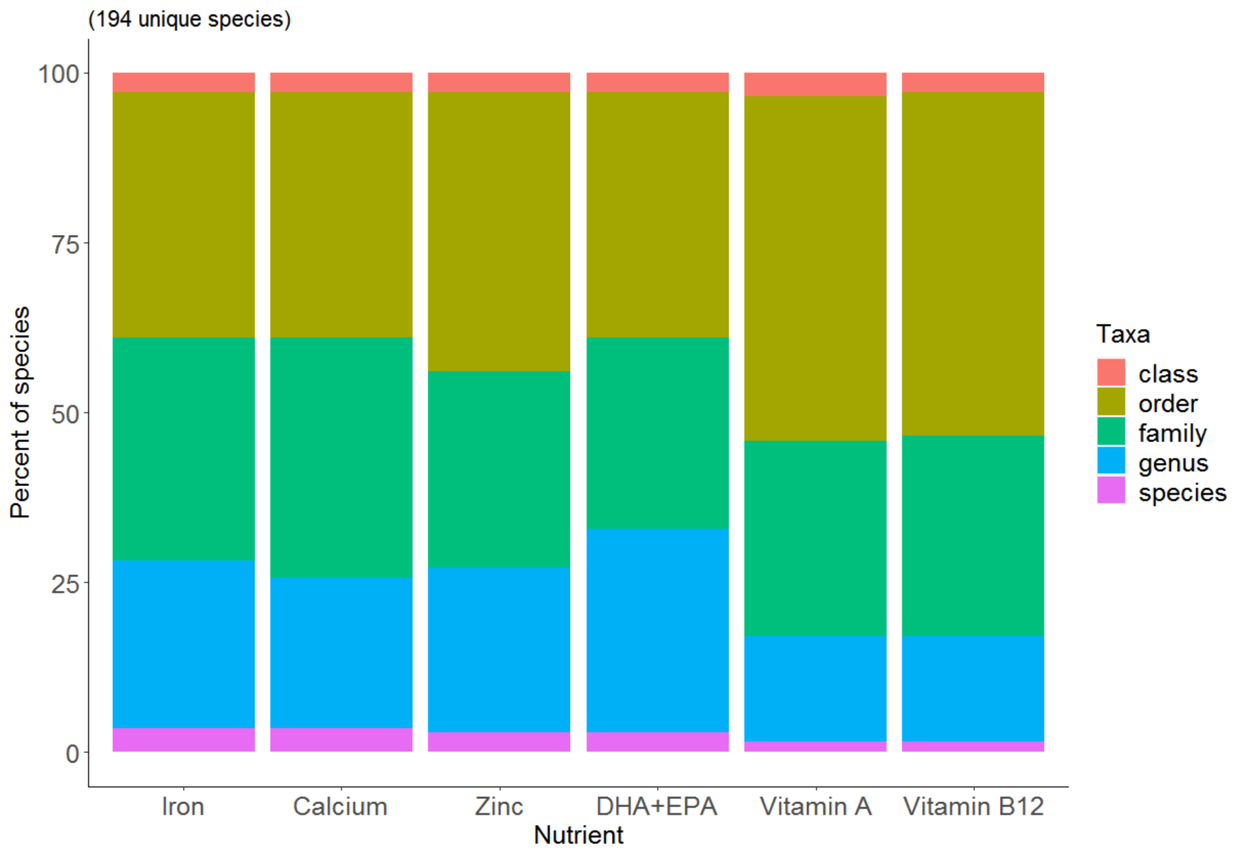

Fig. S6 - Total number of species per nutrient and criteria used to fill nutritional values from the Aquatic Foods Composition Database (AFCD). For all nutrients, there are a total of 194 unique species derived from Sea Around Us reef catch data.

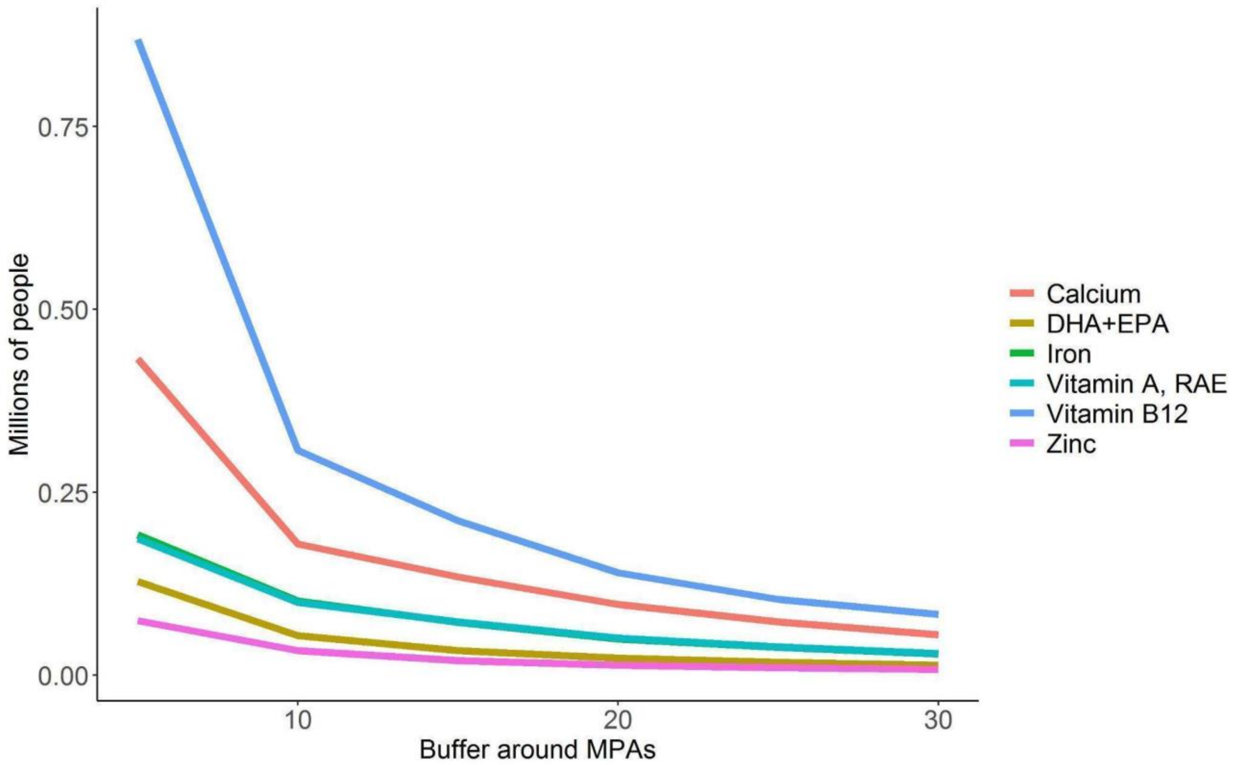

Fig. S7 - Sensitivity of main results to assumed population size within a buffer around non-MPA reefs. The Y axis represent the total number of people transitioning from inadequate to adequate nutrient intake.

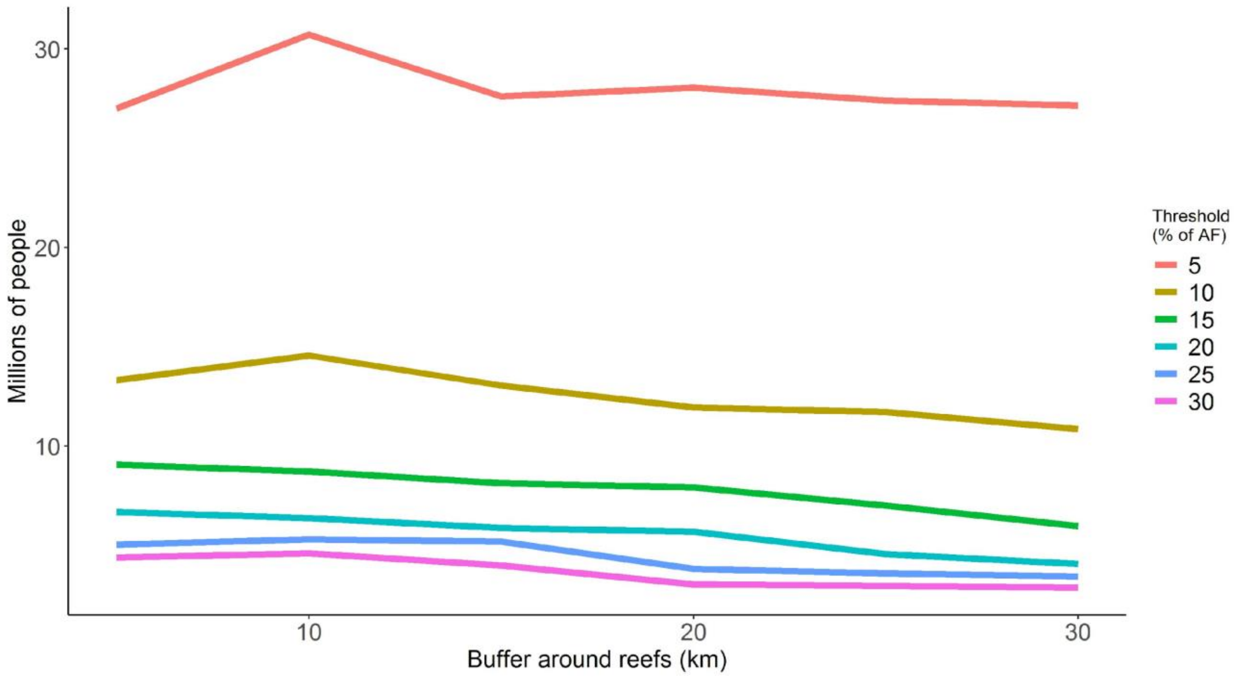

Figure S8 - Sensitivity of total number of people supported by MPAs to assumed population size within a buffer around non-MPA reefs and contribution threshold. The Y axis represent the total number of people supported by MPA expansion.

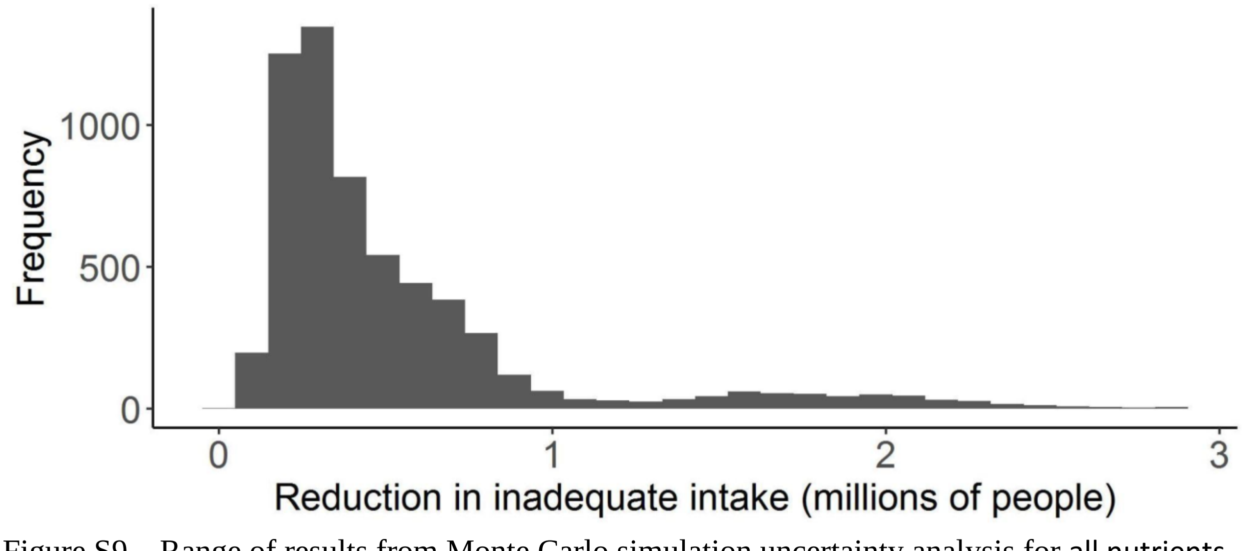

Figure S9 – Range of results from Monte Carlo simulation uncertainty analysis for all nutrients combined (iron, EPA+DHA, calcium, zinc, and vitamins A and B_{12}).

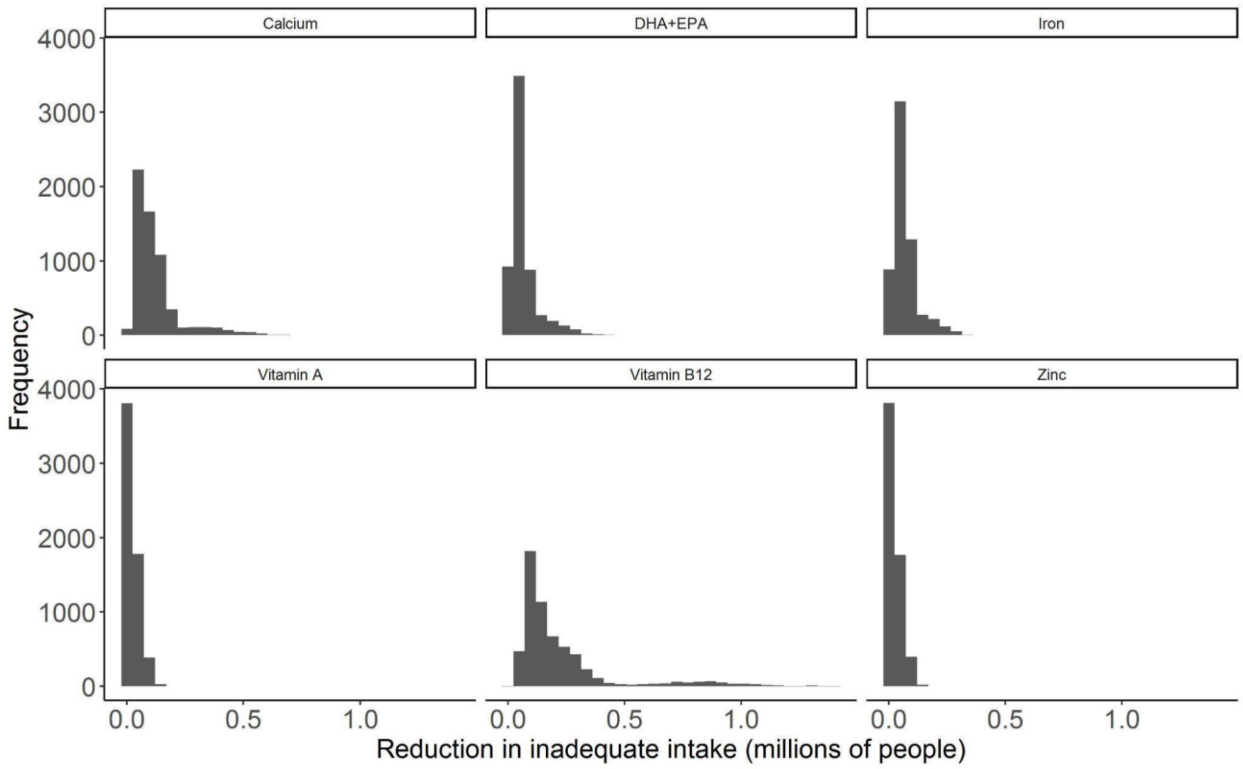

Figure S10 – Range of results from uncertainty analysis using Monte Carlo simulation for each assessed nutrient.

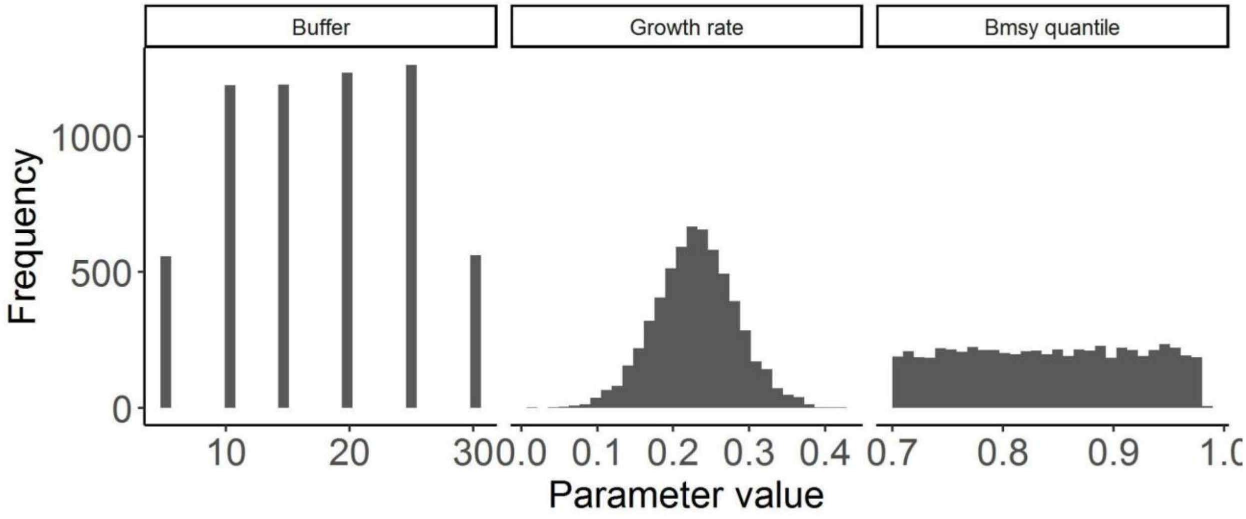

Figure S11 – Parameter values used in Monte Carlo simulation for uncertainty estimates.

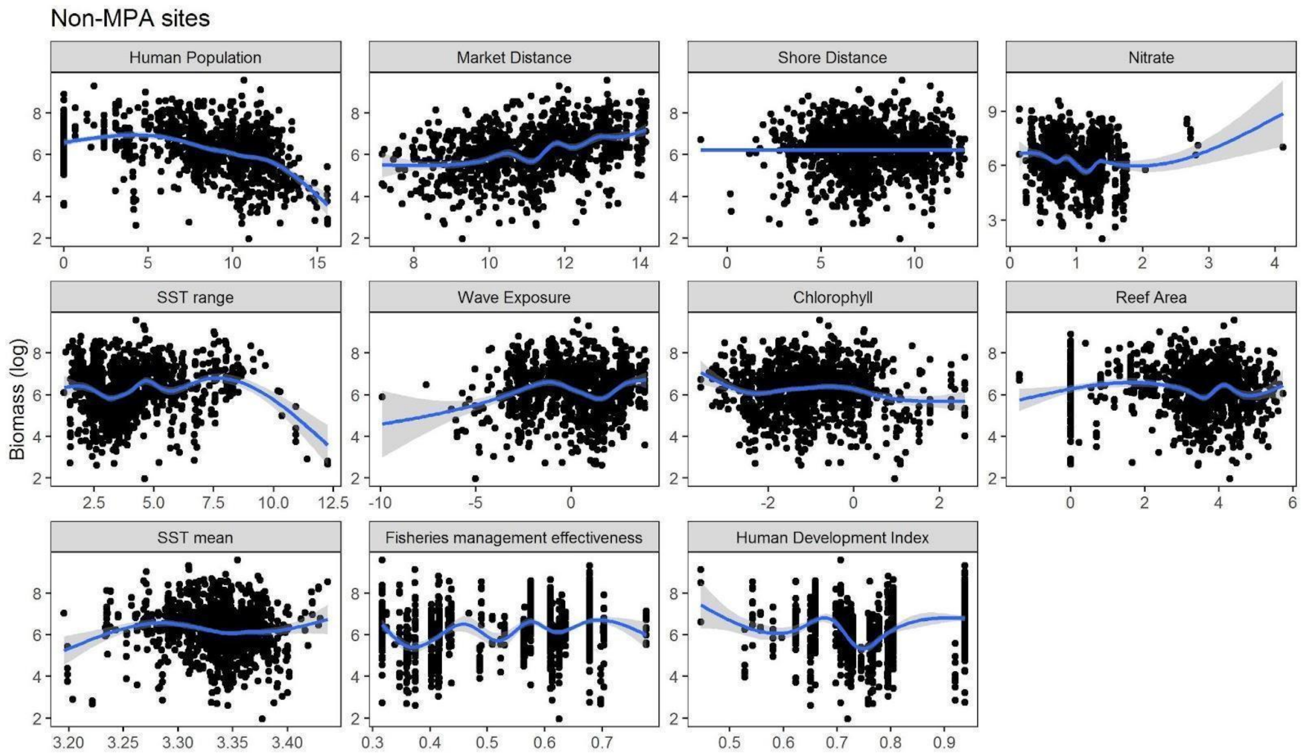

Figure S12 - Relationship between biomass density (log kg/ha) and covariates used in the predictive model for all non-MPA sites.

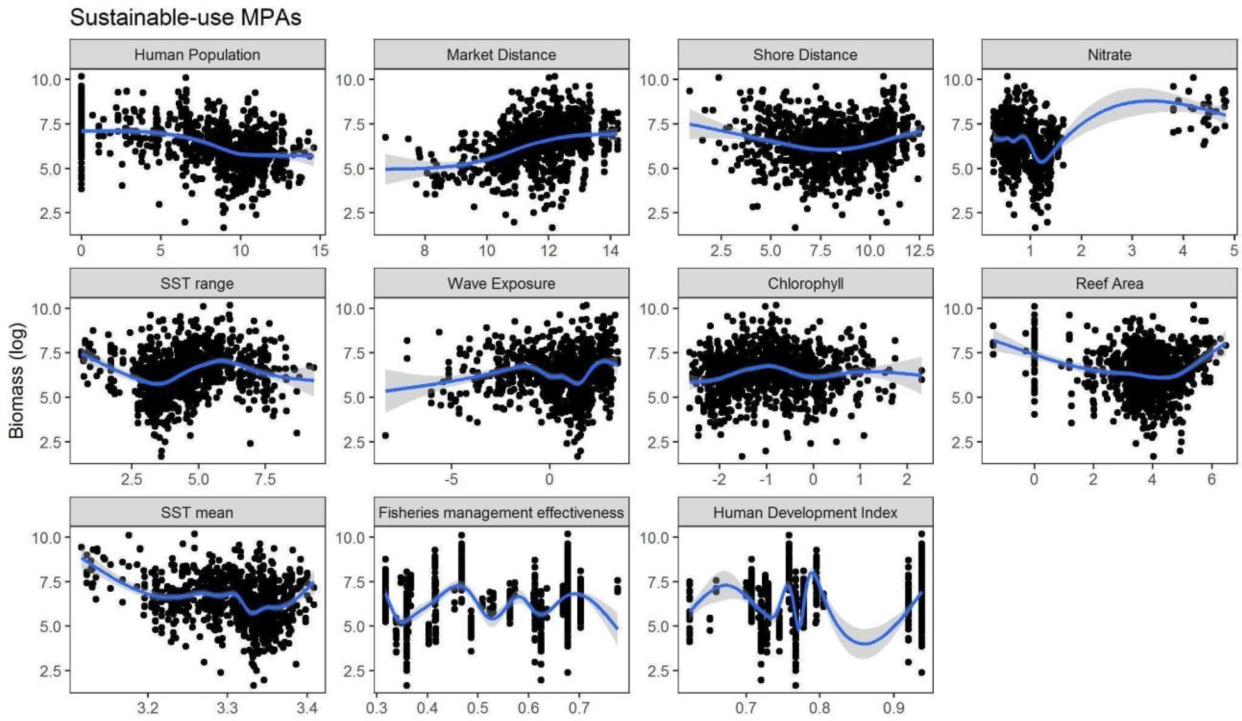

Figure S13 - Relationship between biomass density (log kg/ha) and covariates used in the final model for all sustainable-use MPA sites.

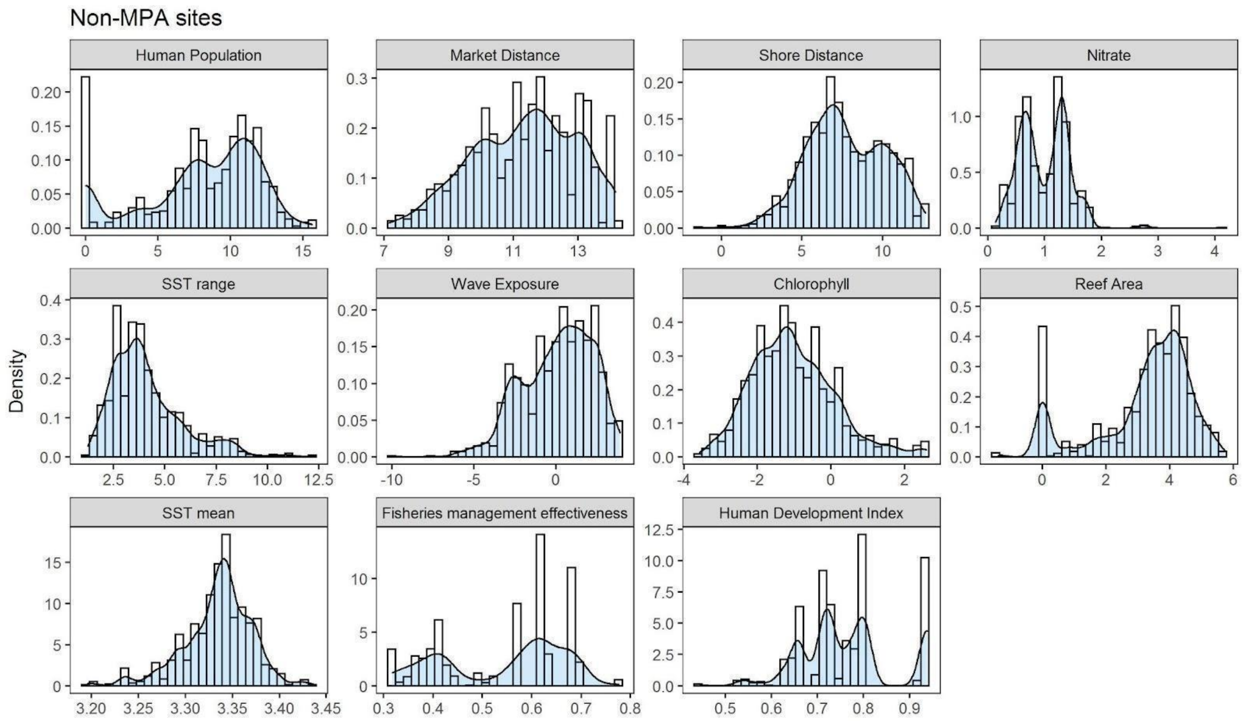

Figure S14 - Density distribution of covariates used in the final model in all non-MPA sites.

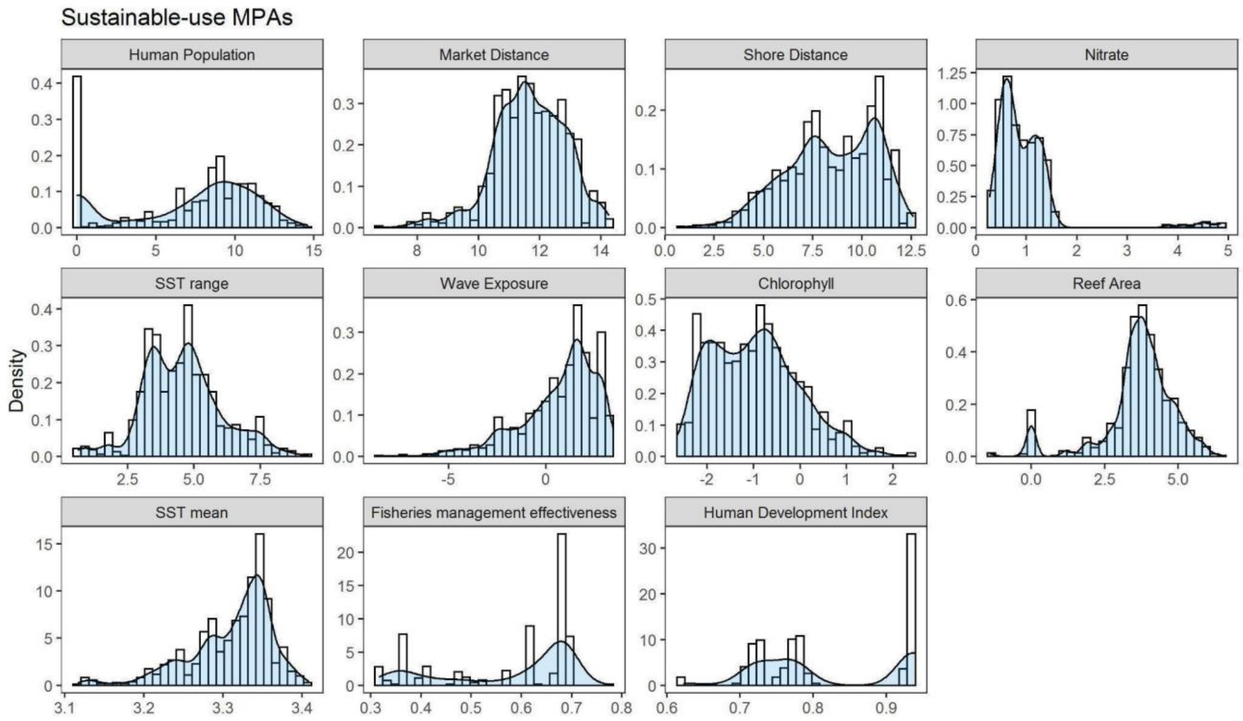

Figure S15 - Density distribution of covariates used in the final model in all sites within sustainable-use MPAs.

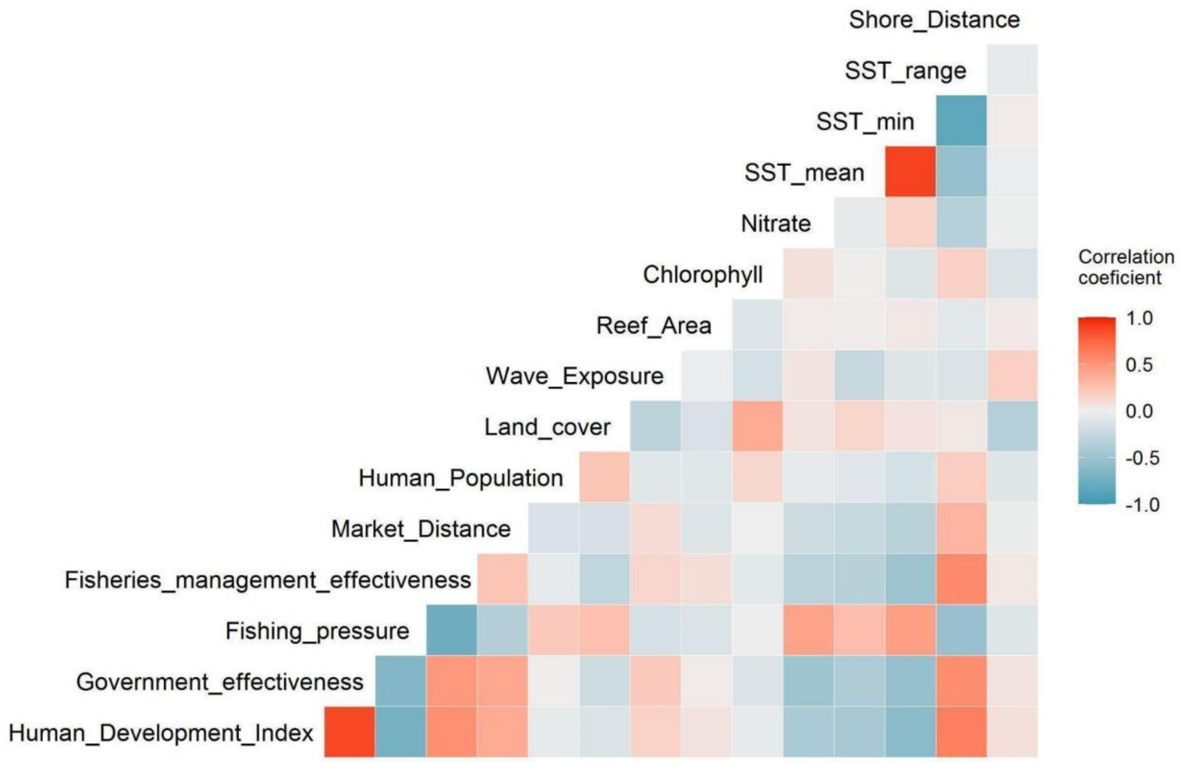

Figure S16 - Pearson correlation plot of model covariates.

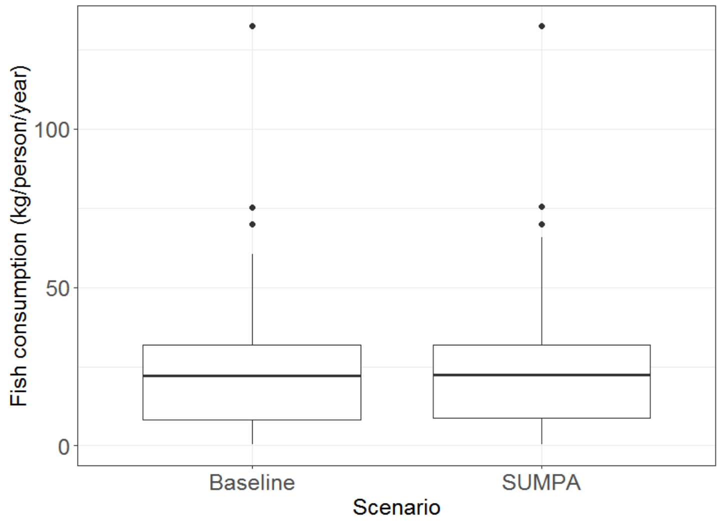

Figure S17 - Comparison of original intakes versus estimated intakes with implementation of sustainable-use MPAs

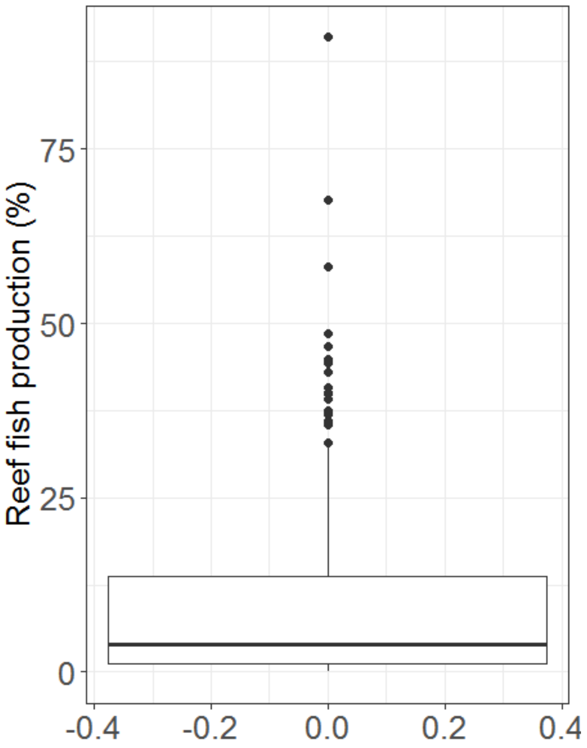

Figure S18 - Contribution of reef fish to national aquatic food production within coral reef countries.
